# Retinol and α-Tocopherol Levels in the Serum and Subcutaneous Adipose Tissue of Newly Diagnosed Basal Cell Carcinoma Patients

**Published:** 2019-10

**Authors:** Ehsan GHAEDI, Fatemeh RAHROVANI, Mohammad Hassan JAVANBAKHT, Amir-Hooshang EHSANI, Ali ESRAFILI, Hamed MOHAMMADI, Mahnaz ZAREI, Mahmoud DJALALI

**Affiliations:** 1. Students' Scientific Research Center, Tehran University of Medical Sciences, Tehran, Iran; 2. Department of Cellular and Molecular Nutrition, School of Nutritional Sciences and Dietetics, Tehran University of Medical Sciences, Tehran, Iran; 3. Department of Dermatology, Autoimmune Bullous Diseases Research Center, Razi Hospital, Tehran University of Medical Sciences, Tehran, Iran; 4. Department of Environmental Health Engineering, School of Public Health, Iran University of Medical Sciences, Tehran, Iran; 5. Department of Community Nutrition, School of Nutrition and Food Science, Isfahan University of Medical Sciences, Isfahan, Iran; 6. Students' Research Committee, Isfahan University of Medical Sciences, Isfahan, Iran

**Keywords:** Skin neoplasms, Basal cell carcinoma, Vitamin A, Alpha-tocopherol, Subcutaneous fat

## Abstract

**Background:**

Nonmelanoma skin cancers are the most frequently occurring skin cancers. Vitamin A is involved in epithelial cell differentiation and may control skin tumor development. Vitamin E is a powerful lipophilic antioxidant that can quench and scavenge reactive oxygen species. However, there is little consistent evidence considering micronutrients and the development of basal cell carcinoma (BCC). Therefore, we aimed to investigate the possible difference between retinol and α-tocopherol in BCC patients and controls in Iranian population.

**Methods:**

This case-control study was conducted on adults with newly diagnosed BCC referred to Razi Hospital, Tehran, Iran in 2015. Serum and subcutaneous fat tissue retinol and α-tochopherol were measured by HPLC method.

**Results:**

Overall, serum retinol level was lower significantly in BCC patients (0.237±0.01 μg/ml) in comparison with control group (0.27±0.02 μg/ml, *P*-value: 0.038). However serum α-tocopherol level was not significantly different between BCC patients (4.41±0.33 μg/ml) and control subjects (4.06±0.35 μg/ml, *P*-value=0.18). Sub-cutaneous adipose tissue retinol was lower significantly in BCC patients (38.60±3.30 ng/mg) compared with control group (54.78±3.49 ng/mg, *P*-value=0.002). Furthermore, results revealed lower subcutaneous adipose tissue α-tocopherol in BCC patients (4.41±0.33 μg/ml) in comparison with control group (4.06±0.35 μg/ml, *P*-value=0.18).

**Conclusion:**

Skin tissue concentration of retinol and α-tocopherol and serum retinol level was lower in BCC patients in comparison with control group but serum α-tocopherol was not different between groups.

## Introduction

Nonmelanoma skin cancer (NMSC) including Basal cell carcinoma (BCC) and squamous cell carcinoma (SCC) are the most frequently occurring skin cancers in white populations and incidence rates have increased by 5-fold worldwide in the last 3 decades (1, 2). Skin cancer is the most common cancer and BCC is the frequent morphologic type of skin cancer in Iran (3, 4). BCCs are slow-growing tumors which cause local tissue damage and are the most common type of keratinocytic skin cancer (KSC), occurring more frequently than SCC (5).

UV radiation is the main risk factor for NMSC (4). Greater exposure to ultraviolet radiation as a consequence of ozone layer depletion and aging of the population has been suggested as explanation for increased NMSC incidence around the world (6). The probability remains for factors other than sun exposure, including age, sex, skin phototype (I, II), and also diet which influences the development of BCC (6, 7).

Both direct DNA damage because of absorbance of UV radiation and indirect DNA damage contributed to reactive oxygen species (ROS) may lead to mutations and skin cancer (8). Skin also has several defense mechanisms include efficient repair mechanisms and an antioxidant defense system (9). The antioxidant levels play an important role in the pathogenesis and treatment of skin carcinomas. Cellular communication, cell receptor functioning, DNA repair systems and cell proliferation pathways may be altered because of UV-induced free radicals (8). Several animal and in vitro studies provided enough evidence that dietary antioxidants may play a crucial role in skin cancer prevention (5). Vitamin A is involved in epithelial cell differentiation and may control skin tumor development (10). In vivo models have reported that retinoids decrease tumor size and improve survival in skin cancer (11). Vitamin E is a powerful lipophilic antioxidant that can quench and scavenge reactive oxygen species (10). Tocopherol also has several other functions, including inhibition of protein kinase C (PKC) activity and suppression of tumor angiogenesis (12). Like previous epidemiological studies considering associations between micronutrients such as retinoids and cancer risk, the role of antioxidant level for BCC development was not consistent (13, 14). Despite previous studies which reported no significant relationship between either plasma retinol concentrations or intake (6, 8, 15), inverse association of serum retinol have also been reported in keratinocytic cancer risk (8) and other cancers including lung (16), breast (17) and gastric cancer (18). Furthermore, the evidence is insufficient for effect of vitamin E in nonmelanoma skin cancer risk. Some studies found no relationship between vitamin E intake and BCC risk but others found inverse association between vitamin E intake and BCC risk (5, 8).

Although previous studies reported altered vitamin metabolism in different cancers, to best of our knowledge subcutaneous adipose tissue level of retinol and alpha-tochopherol in BCC patients have not been investigated. Here, we aimed to investigate the possible difference between retinol and α-tocopherol in BCC patients and control subjects in Iranian population.

## Materials and Methods

### Study Population

We carried out a hospital-based case-control study on new cases of BCC patients in Razi Hospital, Tehran, Iran in 2015. Forty BCC patients with histopathologically confirmed disease were included in present study.

The biopsy and blood samples of the cases were collected following the ethical committee of TUMS approval of the study protocol (Ethical approval number: IR.TUMS.REC.1394.635). All of the study procedures performed according to guidelines in the Helsinki Declaration. Informed consent was obtained from all patients.

The control subjects were randomly selected from first-visit outpatients who visited Razi Hospital without any history of skin cancer. Exclusion criteria for both groups are taking any supplement which contained vitamins, previous systemic disorder other than skin cancer like diabetes mellitus, cardiovascular diseases and the history of treatment. Cases and controls both were matched on Body Mass Index (BMI) for controlling possible confounding factors.

### Data Collection and Sample acquisition

All of patients completed a standardized, questionnaire pertaining to basic demographic data, medical and drug history, etc., through face to face interview by qualified interviewer. Weight and height were calculated by Digital seca 700, based on approved procedures for measurement. BMI was calculated as weight in kg divided by height in meters squared.

Five milliliters blood samples were acquired from antecubital vein after 8–12 h overnight fasting and then were centrifuged in 3000 RPM and the overlaying serums were kept in −80 refrigerators until analytical measurements. The subcutaneous fat tissue samples were incised after removal or biopsy of lesions. The fat tissue samples were kept in −80 freezers until analytical measurements.

### Dietary assessment

Dietary assessment performed through two 24-hour dietary recalls by nutritionist help in two randomly selected days (week/day) and then were analyzed using Nutritionist IV software (First Databank, San Bruno, CA, USA) adjusted for Iranian foods.

### Materials

Ethanol, methanol and hexane were HPLC grade from Sigma-Aldrich. (St. Louis, MO, USA). Moreover, sodium dodecylsulfate, sodium monobasic phosphate (NaH 2 PO 4), dihydrate disodium ethylenediamine tetraacetic acid, retinyl acetate (Na 2 EDTA. 2H 2 O) were purchased from Sigma Company (St. Louis, MO, USA).

### Sample preparation and HPLC analysis:

Each sample was added in a tube which contained 2 mL of a buffer (pH 7.0). The buffer consisting of NaCl 130 mM in H 2 O, Na 2 EDTA. 2H 2 O (1 mM), and NaH 2 PO 4 (10 mM), 50 μL butyl hydroxyl toluene as an antioxidant (1 mg/mL in ethanol), and 1 mL sodium dodecylsulfate (0.1 M). The sample was homogenized for 5 min and after addition of 2 mL ethanol vortexed for 15 sec. Two milliliters of hexane were added followed by vortexing for 15 sec. The final mixture was centrifuged for 15 min at 500 rpm. Then, 1.5 mL of the hexane layer was transferred to a vial, and the solvent was evaporated under nitrogen stream. The residue was dissolved in 0.2 mL methanol, reagent alcohol (ethanol/2-propanol 95/5) 1: 1, vortexed and was kept in storage at −80 °C, until injection to the HPLC system. Because of light sensitivity of retinol and a-tochopherol during both extraction procedures, the samples and solutions were shielded from light.

The sample injected in reverse phase (RP) high-performance liquid chromatographer (LKB, Pharmacia, Russia) with C18 NovaPak column (L, 125nm; ID, 3mm; dP, 3μm) for quantification of variables. Quantitation was performed by a standard curve equation in a range of 0.05–10 μ M. Tests with standard fresh solutions were frequently injected during analysis.

### Statistical Analysis

Distribution of dermatological characteristics and food intakes were compared between BCC patients and controls by Kolmogorov-Smirnov test. Test for continuous variable and dietary intakes between case and control groups were compared using Independent t-test. Means of dietary intakes were adjusted for age and gender. All values expressed as mean ± SEMs. If *P*-value was less than 0.05, it was considered significant. All statistical analysis were performed using SPSS ver. 17 (Inc., Chicago, Illinois, USA).

## Results

Demographic characteristics of cases and controls were shown in Table 1. There were no statistically significant differences for any of the demographic characteristics between BCC and control groups. Dietary assessment analysis revealed that there were no statistical significant differences between groups as shown in Table 2. Energy intake, all of macronutrients and micronutrients especially retinol and α-tocopherol were not different in patients and control groups.

**Table 1:** Demographic characteristics and medical history of control and Basal Cell Carcinoma group^#^

***Demographic characteristics***		***BCC***	***Control***	P value*
Sex	Male (%)	28 (70%)	21 (52.5%)	0.18^**^
	Female (%)	12 (30%)	19 (47.5%)	
Age (years)		57.76±1.56	54.05±1.16	0.06*
Weight (kg)		72.39±1.94	72.48±2.46	0.98*
Height (cm)		167.76±1.59	166.30±1.36	0.49*
BMI (kg/m^2^**)**		26. 02±0.74	25.74±0.67	0.78*

# Mean ± SE

* *P* reported based on Independent Sample *t*-test

** *P* reported based on Chi-Square test.

**Table 2:** Energy, macronutrients and micronutrients intakes of BCC subjects and control group^#^

***Variable***	***BCC***	***Control***	P value*
Total energy (kcal)	2537.65±146.68	2246.55±234.98	0.27
Total carbohydrate (g)	327.32±23.78	320±27.55	0.86
Total protein (g)	68.85±3.33	68.90±4.99	0.99
Total fat(g)	80.82±6.02	85.49±8.09	0.65
SFA (g)	20.22±1.91	17.57±1.55	0.36
MUFA (g)	32.00±3.98	29.63±2.84	0.62
PUFA(g)	30.41±3.36	36.06±6.39	0.39
Cholesterol (mg)	115.37±39.94	120.42±11.65	0.74
Oleic fat (g)	19.56±2.06	23.24±2.2	0.26
Linolenic (g)	2.47±1.33	1.33±0.39	0.54
Linoleic (g)	28.47±3.19	34.27±21.18	0.37
DHA (g)	0.02±0.01	0.001±0.00	0.39
EPA (g)	0.01±0.00	0.00±0.00	0.43
Dietary Fiber (g)	23.87±9.50	7.94±1.42	0.22
Vitamin C (mg)	67.21±12.89	33.61±9.19	0.08
Vitamin D (μg)	7.58±5.13	1.61±.42	0.39
Selenium (mg)	0.22±0.15	0.04±0.00	0.38
Magnesium(mg)	255.19±14.37	234.93±28.88	0.48
Calcium(mg)	654.26±81.05	473.95±65.62	0.14
Zinc (mg)	8.38±0.53	9.27±0.81	0.34
Alpha tocopherol (mg)	12.63±1.60	14.36±2.86	0.57
Beta carotene (μg)	613.55±186.05	538.35±220.61	0.80

# Mean ± SE

* *P* reported based on Independent Sample t-test

SFAs= saturated fatty acids, PUFAs= polyunsaturated fatty acids, MUFAs= monounsaturated fatty acids

**Fig. 1:**
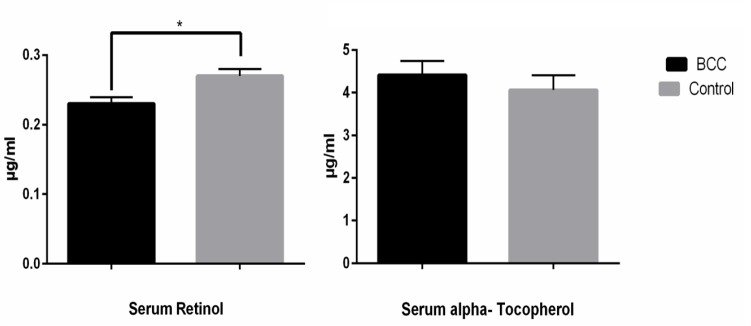
Retinol and alpha-Tochopherol serum concentration in BCC patients and control subjects

**Fig. 2:**
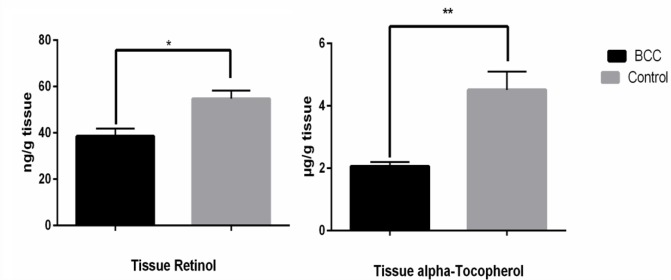
Retinol and Alpha-Tochopherol tissue concentration in BCC patients and control subjects

Fig. 1 shows the level of serum Retinol and alpha-Tochopherol in BCC patients and their age-matched controls. Serum retinol was significantly (*P*=0.002) lower in cancer patients (0.237±0.01 μg/ml) than in their matched controls (0.27±0.02 μg/ml). There was no significant difference (*P*=0.18.) in serum α-tocopherol levels. Fig. 2 shows tissue retinol and alpha-tochopherol levels in both groups. Subcutaneous adipose tissue from cancer patients contained significantly lower retinol (P=0.002) and α-tocopherol (P<0.001) levels than the control tissue samples (Retinol 38.60±3.30 ng/mg tissue in BCC group against 54.78±3.49 ng/mg tissue in control group and α-tocopherol 2.06±0.14 μg/g tissue in BCC group gainst 4.51±0.59 μg/g tissue in control group).

## Discussion

In the present study, newly diagnosed BCC patients have lower α-tochopherol and retinol concentration in skin specimen and lower serum retinol concentration in comparison with control group. However, serum α-tochopherol was not significantly different between two groups. These results obtained while dietary intake also showed no statistical difference between patients and control groups.

Previous studies of skin cancer and diet have failed to indicate beneficial effect of retinol and its derivatives for prevention of new NMSC (19). However, intake of retinol is significantly associated with reduced risk of melanoma (20). Previous case-control and cohort studies found no relationship between either plasma retinol concentration or intake and risk of NMSC (6, 8, 15). On the contrary, higher serum retinol levels among patients with BCC compared with controls have been reported (8). Here we show lower concentration of serum retinol in BCC patients. Inverse association of retinol has also been reported in other cancers including lung (16), breast (17) and gastric cancer (18). Furthermore, in one case-control study and one nested case control study in BCC and SCC patients, there was an inverse association between serum retinol and keratinocytic cancer risk (8). An inverse association was reported with use of vitamin A supplements in the hospital-based case-control study in BCC patients (8). Retinoids have been indicated to prevent NMSC in high-risk patients such as those with Xeroderma Pigmentosaand organ transplants (21). Different study designs, different study population as well as method of retinol assessment might have contributed to the inconsistency of published studies.

The evidence for a protecting effect of vitamin E in NMSC is weak. Although some studies found no relationship between either vitamin E serum level or intake and risk of BCC (5), other studies found inverse association between vitamin E intake and BCC risk (8). In addition, one cohort study showed a significant positive relationship between vitamin E intake and BCC risk (22). A double-blind clinical trial found no significant difference in clinical or histological response to Ultraviolet B (UVB) in patients receiving 6 months of oral vitamin E supplement compared with placebo (23). We found no significant difference in the serum level of α-tochopherol in BCC patients and control subjects.

BCCs have a long-term induction period and it is still probable that diet act at earlier stages of keratinocytic cancer development (24, 25). A probable explanation for the lack of association between dietary factors and BCC is that the related period of dietary exposure was not measured (26, 27). Actually, most of serum/plasma biomarkers are hypothesized to indicate dietary exposure over the short term despite dietary intake estimation tended to represent a longer-term estimation of habitual intake (28).

We investigated also subcutaneous adipose tissue concentration of α-tochopherol and retinol. However, majority of studies are focused on enzymatic antioxidants such as glutathione peroxidase or glutathione and superoxide dismutase (29, 30). Considering all the mechanisms during carcinogenesis and its progression, stimulation of lipid metabolism occur because of peroxidation of membrane lipids and phospholipids (31) which can increase lipophilic environment. The increasing lipophilic environment in BCC not only lead to reduction in the solubility of hydrophilic antioxidants but also lipophilic environment encircles the lipophilic antioxidants (α-tocopherol and retinol) and prevents them from reacting as skin protectors (32). Moreover, constant concentrations of lipophilic antioxidants are ensured through regeneration by taking hydrogen atoms from thiols and vitamin C, so they could protect the skin from oxidative stress (32). However, lipid peroxidation and amyloid-like protein formation causes more lipophilic environment. In these circumstances the solubility of hydrophilic antioxidants especially Vitamin C decreased and consequently reduces the action of lipophilic antioxidants. Oxidative stress also can influence concentration of lipophilic antioxidants. In vitro experiments indicated that vitamin E concentrations influence the proteins' physiological structure like α-helix and β-sheet (33). In spite of oxidative stress nature of skin cancer, protective mechanism of the body does not react under these conditions (32). Our results indicated lower concentration of these fundamental antioxidants, however we have not measured their activity, it must be decreased based on what is mentioned.

Mechanisms by which antioxidant nutrients may inhibit carcinogenesis include scavenging of reactive intermediates; inhibition of cell proliferation; induction of cell differentiation; and induction of apoptosis (34). Vitamin A and its derivatives inhibit the activation of the ERK (Extracellular signal-regulated kinases) and JNK MAPK (C Jun N Terminal Kinase Mitogen-Activated Protein Kinase) pathways and the activity of AP-1 (Activator protein 1). Moreover, it can induces cell-cycle arrest through some proteins including cyclin D1/CDK4 (Cyclin-dependent kinase), cyclin E/CDK2, p21 and p27 (35). Alpha-Tocopherol inhibits protein kinase C activity and could directly modulate genes involved in growth, apoptosis and inflammation. Protein kinase C plays an important role in cell proliferation, adhesion, immune responses, and gene expression (36).

Our study had strengths mentioned. The newly diagnosed serum specimens minimized the possible influence of disease progression and treatment. Skin specimen is another strength which allows further interpretation. The third strength of this study is that there were two measures of dietary exposure which were serum biomarkers and recall estimates of dietary intake. Some methodological limitations, however, need clarification. We could not comprise all the potentially confounding factors. For example, considering serum folate reported to influence BCC risk (6) and also other nutrients which not measured. Next, the sample size was a limitation of this study that we cannot examine the sex-specific effects of variables.

## Conclusion

Overall, BCC patients have lower subcutaneous adipose tissue concentration of retinol and α-tochopherol. Although serum retinol was lower in BCC patients in comparison with control subject our results did not show significant differences in α-tochopherol in cancer patients. This study, along with previous studies provides further evidence for alteration of retinol and α-tocopherol level in BCC. However, the results must be evaluated with caution because of small sample size. Although possible protective role of some antioxidants has been reported in BCC due to lack of studies which investigating subcutaneous adipose tissue level of antioxidant in BCC patients, there is currently insufficient evidence to draw conclusions. Future studies should focus on subcutaneous adipose tissue concentration of antioxidants to clarify new points in this disease. Furthermore high-quality comprehensive studies with larger numbers of cases and different population are necessary for investigating the possible effect of sex or other confounding factors.

## Ethical considerations

Ethical issues (Including plagiarism, informed consent, misconduct, data fabrication and/or falsification, double publication and/or submission, redundancy, etc.) have been completely observed by the authors.
